# Chinese Herb Injections in the Adjuvant Treatment for Ulcerative Colitis: A Network Meta-Analysis of Randomized Controlled Trials

**DOI:** 10.1155/2022/3166416

**Published:** 2022-04-22

**Authors:** Ziyang Zhou, Hao Chen, Yingkai Shen, Hailiang Huang

**Affiliations:** ^1^College of Traditional Chinese Medicine, Shandong University of Traditional Chinese Medicine, Jinan 250355, China; ^2^College of Rehabilitation Medicine, Shandong University of Traditional Chinese Medicine, Jinan 250355, China

## Abstract

Ulcerative colitis refers to an inflammatory disease lasting for a long time, which affects the colon. In China, injections of traditional Chinese herbs have been generally combined with traditional Western medicines such as mesalazine and sulfasalazine to treat ulcerative colitis. Nevertheless, the safety and efficacy exhibited by different CHIs for treating UC remains controversial. Therefore, a network meta-analysis method was employed in this study for the assessment of the effect and safety exhibited by CHI for treating UC. Seven English and Chinese databases were searched for relevant randomized controlled trials (RCTs) from the time of database creation to December 30, 2021. An assessment was conducted for the included RCTs' quality with the use of the Cochrane risk offset assessment device, and this study processed the data with the use of Review Manager 5.3 or Stata16.0 software. On the whole, 42 literature with data on 3668 patients were included. The overall response rate, inflammatory factors, recurrence rate, and adverse reactions were evaluated. In comparison with traditional Western medicines-based treatment, CHI integrated with traditional Western medicines presented an overall response rate (*P* < 0.05) and could better reduce the TNF-*α* (*P* < 0.05), IL-6(*P* < 0.05), and IL-8 level rate (*P* < 0.05) while better increasing the IL-10 level rate (*P* < 0.05). Besides, adverse reactions of CHI integrated with traditional Western medicine had a lower incidence (*P* < 0.05), and no significant distinction was identified in recurrence rate levels between the two interventions. CHI has some efficacy for treating UC. Xiangdan injection, Shenmai injection, Shengmai injection, and Danshen injection may be the most effective CHI. Nevertheless, more multicenter randomized controlled double-blind trials with great quality and large samples are required for research confirmation. Trial Registration: the registration was made for the protocol of this network meta-analysis in PROSPERO with ID CRD42021251429.

## 1. Introduction

Ulcerative colitis refers to an idiopathic disease-causing inflammation that lasts for a long time and impacts the colon. Adults with the age from 30 to 40 years most suffer from ulcerative colitis, which causes their disability [1, 2]. Epidemiological data demonstrate that ulcerative colitis does not show sex predominance [3–5]. The onset of ulcerative colitis happens most significantly between the ages of 30 and 40 years. [4, 6]. Ulcerative colitis has rising incidence and prevalence over time in the globe [7]. Most patients with ulcerative colitis receive treatment by using pharmacological therapy for initially inducing remission and subsequently maintaining corticosteroid-free remission. In terms of mild-to-moderate UC, oral and rectal 5-aminosalycilates have been extensively applied. In accordance with moderate-to-severe colitis, medication types comprise the Janus kinase inhibitor with small molecules, biological agents that target tumor necrosis factor and integrin, and thiopurine [8]. Nevertheless, many traditional drugs for treating UC produce adverse reactions or complications while exerting efficacy. For instance, mesalamine can effectively induce and keep remission [9]. On the other hand, this drug has more severe adverse reactions. Representative negative influence exerted by mesalamine cover paradoxical reaction worsening diarrhea and drug-induced interstitial nephritis with 0.2% risk [10]. Though corticosteroids can effectively induce remission, they have correlations to many complications, many of which are often irreversible [11, 12]. Therefore, it is urgent to discover a treatment for UC with good safety and efficacy.

For treating UC, the main advantage of traditional Chinese medicine is that it can maintain remission for a long time and reduce the recurrence rate [13]. Chinese herbal injection (CHI) refers to a type of novel preparation exhibiting great biological availability and great curative influence [14]. It is an innovative application of dosage, combining traditional Chinese medicine theory with modern scientific technology. At present, CHI integrated with traditional Western medicine is extensively employed to clinically treat UC and has achieved good efficacy. Samuel Wei et al. [15] carried out the meta-analysis for the safety and effect exhibited by Danshen injection integrated to sulfasalazine or mesalazine for treating UC. The results reflected that Danshen injection could significantly improve the clinical effective rate of UC and reduce the recurrence rate compared with Western medicine. Moreover, it can better improve coagulation function, control the inflammatory response, and down-regulate TNF-*α*, IL-8, and IL-6 serum levels in patients. The clinical observation of Liang Xuan [16] revealed that Shengmai injection integrated with mesalazine, compared with mesalazine alone, was more effective and could better reduce the scores of abdominal pain, diarrhea, bloody stools, and tenesmus. Zhu Bingxi [17] discovered that Danshen injection had a protective effect on the mucosa of ulcerative colitis triggered by acetic acid within rats, and the mechanism may be achieved by up-regulating superoxide dismutase (SOD) and downregulating the biological level of malondialdehyde (MDA), indicating that Danshen injection can help scavenge oxygen free radicals. Although various CHIs have acceptable efficacy for treating UC, the effects and safety exhibited by a single CHI integrated to traditional drugs for treating UC were only reported in currently published literature. To date, no meta-analysis comparing different CHIs in combination with traditional drugs for treating UC has been published. Therefore, this paper has an aim at drawing an indirect comparison of the effects and safety exhibited by various CHIs integrated with traditional drugs for treating UC with a network meta-analysis method to lay a more solid basis for clinically treating UC.

We present the following article in accordance with the checklist of the PRISMA extension for network meta-analysis.

## 2. Materials and Methods

### 2.1. Eligibility Standards

#### 2.1.1. Inclusion Standards

1. Study type. It was a randomized controlled trial of CHI integrated with traditional Western medicine for treating UC. Publication languages were limited to Chinese and English.
2. Study subjects. For patients definitely diagnosed with ulcerative colitis, the diagnostic standards indicate the Consensus Opinions on the Diagnosis and Treatment of Inflammatory Bowel Disease issued by the Chinese Medical Association in 2018 [18] without limiting their nationality, age, gender, ethnicity, and course of the disease.
3. Intervention measures. The control received the treatment by using traditional western medicine. The experimental group received the treatment by using CHI or CHI integrated with traditional western medicine. All the covered literature should report any one of the primary or secondary outcome indicators. The primary outcome indicator was the overall response rate. The secondary outcome indicators were as follows: inflammatory factors, recurrence rate, and incidence of adverse reactions.

#### 2.1.2. Exclusion Standards

1. The treatment group is CHI integrated with other treatment methods other than traditional Western medicine
2. Repeated publication of literature
3. Unable to extract data or missing data of literature conference literature
4. Non-RCT literature, such as network meta-analysis, meta-analysis, systematic reviews, reviews, theoretical literature, famous medical practices, animal experiments, case-control literature, and cohort literature

### 2.2. Types of Outcome Measures

In this study, primary and secondary outcome indicators were identified under the guidance of this strategy, based on four versions [18–21] of domestic and international clinical guidelines and expert consensus on ulcerative colitis, combined with the frequency of outcome indicators in the articles.

The main outcome measures were as follows: (1) the overall response rate, which refers to the Chinese Medical Association 2018 Consensus Opinions on the Diagnosis and Treatment of Inflammatory Bowel Disease [18]. The main reference standards included the following: (1) significantly effective: the clinical symptom disappeared, and colonoscopy suggested the mucosa to be approximately normal or no active inflammation; (2) effective: the clinical symptoms basically disappeared, and colonoscopy demonstrated mild mucosal inflammation; and (3) ineffective: rare enhancement in clinical symptom or colonoscopy reexamination. Overall response rate = (number of significantly effective cases + number of effective cases)/(total number of cases) × 100%. The secondary outcome measures were as follows: (2) inflammatory factor (interleukin-10 (IL-10), interleukin-8 (IL-8), interleukin-6 (IL-6), and tumor necrosis factor-*α* (TNF-*α*)); (3) recurrence rate; and (4) incidence of adverse reactions.

### 2.3. Search Strategies

Computerized searches were performed on the databases of CNKI, WAN FANG DATA, VIP, CBM, the Cochrane Library, Embase, PubMed, and Web of Science to obtain published randomized controlled trials (RCTs) of CHI in Ulcerative Colitis (UC) from its inception to December 30, 2021.

A search strategy of subject headings plus free words was used.

Supplementary Table 1 lists the search strategy for the respective database.

### 2.4. Literature Screening and Data Extraction

Relevant literature was searched, and bibliographies were exported in accordance with the search strategy. Endnote X9 software was used to eliminate repeated literature and literature inconsistent with the inclusion standards. The full text of the literature that might meet the inclusion standards was downloaded for determining if it complied with the inclusion standards.

Two authors (Ziyang Zhou and Hao Chen) independently screened, extracted, and cross-checked the literature in accordance with the including and excluding standards. Disagreement was addressed through a discussion with a third investigator (Yingkai Shen).

The data extraction standards included the following: first author, publication time, sample size, gender ratio, mean age, mean disease duration, the number of cases, intervention measures in the test group, intervention measures in the control, course of treatment, outcome measures, and adverse reactions.

### 2.5. Bias Risk Assessment

By complying with the risk of bias tool (Risk of Bias) in Review Manager 5.3 software, two evaluators (Ziyang Zhou and Hao Chen) independently performed a quality assessment for each study from seven perspectives: the blind method for subjects and participants, allocation concealment, random sequence generation, the blind method for result assessment, selective report, incomplete result data, and other deviations. The literature quality was evaluated at three levels, namely, “unclear” (lack of relevant information or uncertain bias), “high” (high bias), and “low” (low bias). Disagreements were resolved by discussing them with a third investigator (Yingkai Shen). The visualization was conducted for risk bias assessment results of the covered literature using Review Manager 5.3 software.

### 2.6. Statistical Investigation

This study employed Review Manager 5.3 software for traditional meta-analysis and literature quality assessment. The odds ratio (OR) and 95% CI acted as effect size indicators for dichotomous variables (overall response rate, recurrence rate, and incidence of adverse reactions). Mean difference (MD) and 95% CI were regarded as effect size indicators for continuous variables (inflammatory factors). All the included literature in this study involved pairwise comparisons, without forming a closed loop. The heterogeneity test was mainly determined by I^2^. If there was no heterogeneity between the study results (I^2^ ≤ 50%), this study employed the fixed-effect model in terms of meta-analysis. If there was heterogeneity among the study results (I^2^ > 50%), the heterogeneity source was further analyzed. After the exclusion of effects exerted by significant clinical heterogeneity, the random-effects model was employed for the meta-analysis. Under a frequency-based random-effects model, STATA16.0 software was adopted for performing a network meta-analysis, in which the study outcome measures were network-analyzed by group commands. Besides, data processing, network evidence plots, funnel plots, forest plots, and ranking of the area under the curve (SUCRA) were completed in turn. The overall ranking of treatments was estimated by calculating the area under the cumulative ranking probability plot (SUCRA) for each method. Moreover, the advantages and disadvantages of the interventions were ranked in accordance with the size of SUCRA. SUCRA = 1 indicated that the interventions were absolutely effective, while SUCRA = 0 suggested that the interventions were absolutely ineffective. The publication bias of the involved literature was evaluated with a funnel plot.

## 3. Results

### 3.1. Literature Search and Screening

**Figure 1:**
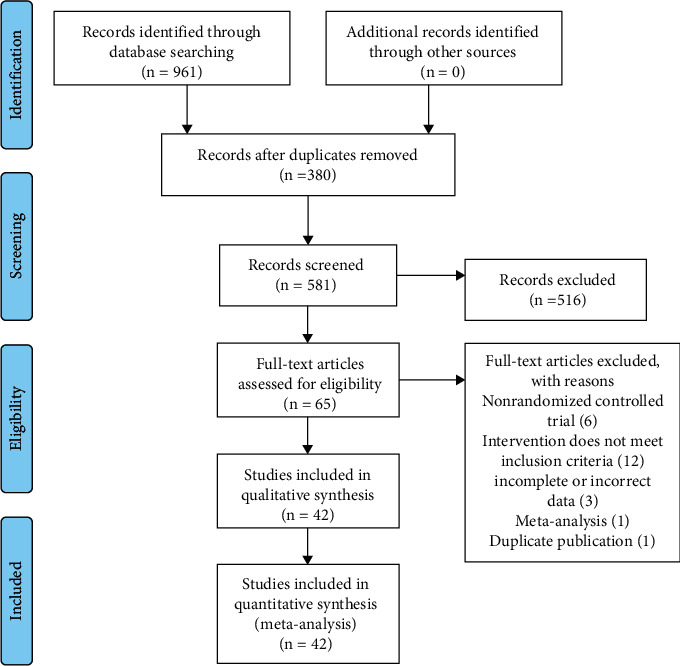
The process of literature filtering.

There were 961 articles initially searched. After the layer-by-layer screening, 42 articles were finally included. The screening of literature is illustrated in Figure 1.

### 3.2. Basic Characteristics of Involved Literature

On the whole, 42 pieces of literature [19, 21–61] which included 3668 patients. Supplementary Material Table 2 gives the basic information of the involved literature.

### 3.3. Bias Risk Assessment of Involved Literature

Regarding random sequence generation, seven pieces of literature employed a random number table for random allocation. One study used the single and double number method for randomization, one study applied randomization in accordance with the date of admission, and two literature studies did not mention randomization. Additionally, no specific method of randomization was reported in the remaining 31 pieces of literature. Concerning randomization concealment, none of the literature mentioned the use of any allocation concealment method, nor the use of the blind method about blinding of the subjects to interventionalists. In terms of blinding the evaluators of the results, one study used a double-blind of pathological findings, while the use of the blind method for the evaluators of the results was not presented in the remaining 41 pieces of literature. For incomplete result data, four pieces of literature had dropout/withdrawal cases, which may affect the true results. Particularly, there was no missing outcome data for the remaining 38 pieces of literature. All literature reported all prespecified outcome measures given selective reporting.

**Figure 2:**
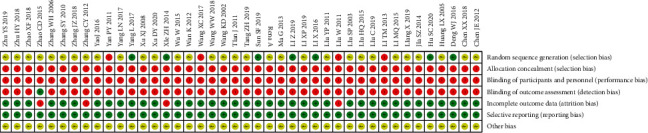
Risk of bias for all RCTs included in this study.

None of the literature reported other sources of bias. The risk of bias assessment of the involved literature is illustrated in Figure 2.

### 3.4. Outcome Indicators

#### 3.4.1. Total Effectiveness Rate

**Figure 3:**
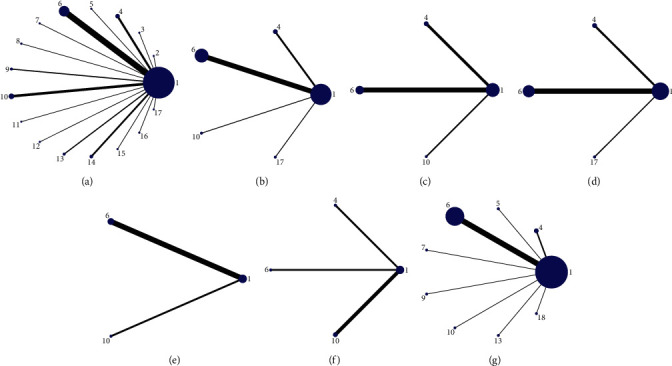
Network diagrams of outcome indicators. (a) Overall response rate; (b)TNF-*α*; (c)IL-6; (d)IL-8; (e)IL-10; (f) recurrence rate; (g) adverse reaction rate. 1: Conventional western medicine, 2: conventional Western medicine + Shenfu injection, 3: conventional Western medicine + Shenqi Fuzheng injection, 4: conventional Western medicine + Danshen powder injection, 5: conventional Western medicine + compound Danshen injection, 6: conventional Western medicine + Danshen injection, 7: conventional Western medicine + compound Kushen injection, 8: conventional Western medicine + Guanxinning injection, 9: conventional Western medicine + Safflower injection, 10: conventional Western medicine + *Astragalus* injection, 11: conventional Western medicine + Shenmai injection, 12: conventional Western medicine + Acanthopanax injection, 13: conventional Western medicine + Shengmai injection, 14: conventional Western medicine + Shuxuening injection, 15: conventional Western medicine + Xiangdan injection, 16: conventional Western medicine + Xuesaitong powder injection, 17: conventional Western medicine + Houttuynia injection, and 18: conventional Western medicine + Angelica injection.

*(1) Evidence Network*. Thirty-seven pieces of literature reported overall response rates involving 17 CHI treatment regimens. The dot size indicates the sample size using the intervention, the line thickness represents the number of RCTs using the two-point treatment intervention, all 17 CHIs denote direct comparisons, and there is no closed-loop formation. The network evidence of the overall response rate is exhibited in Figure 3.

**Figure 4:**
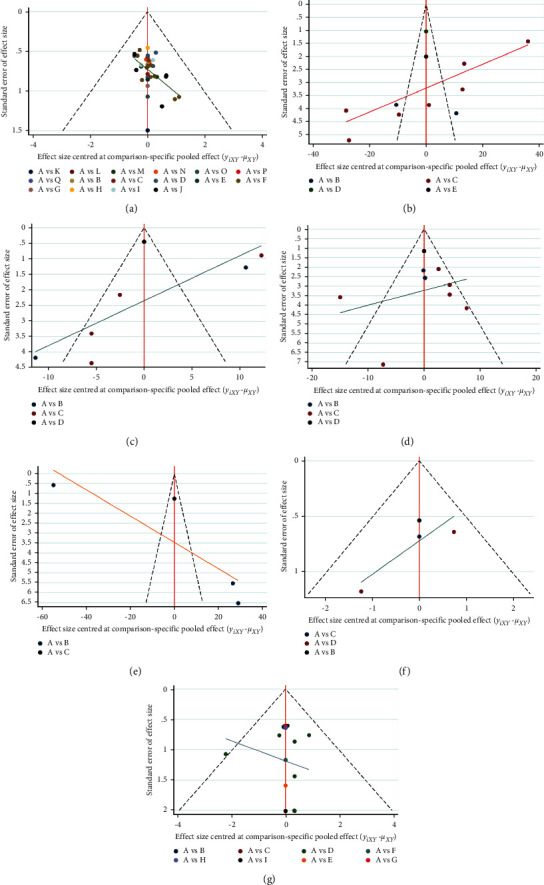
Funnel plot of outcome indicators. (a) Overall response rate; (b) TNF-*α*; (c) IL-6; (d) IL-8; (e) IL-10; (f) recurrence rate; (g) adverse reaction rate. A : Conventional Western medicine, B : conventional Western medicine + Shenfu injection, C : conventional Western medicine + Shenqi Fuzheng injection, D : conventional Western medicine + Danshen powder injection, E : conventional Western medicine + compound Danshen injection, F : conventional Western medicine + Danshen injection, G : conventional Western medicine + compound Kushen injection, H:conventional Western medicine + Guanxinning injection, I : conventional Western medicine + Safflower injection, J : conventional Western medicine + *Astragalus* injection, K : conventional Western medicine + Shenmai injection, L : conventional Western medicine + Acanthopanax injection, M : conventional Western medicine + Shengmai injection, N : conventional Western medicine + Shuxuening injection, O : conventional Western medicine + Xiangdan injection, P : conventional Western medicine + Xuesaitong powder injection, and Q : conventional Western medicine + Houttuynia injection.

*(2) Publication Bias*. The funnel plot of this study revealed that most of the scatter points were located on both sides of the vertical line. They were basically symmetrical and may have had a certain degree of publication bias. The funnel plot of the overall response rate of 17 CHIs integrated with traditional Western medicine for treating UC is presented in Figure 4.

**Figure 5:**
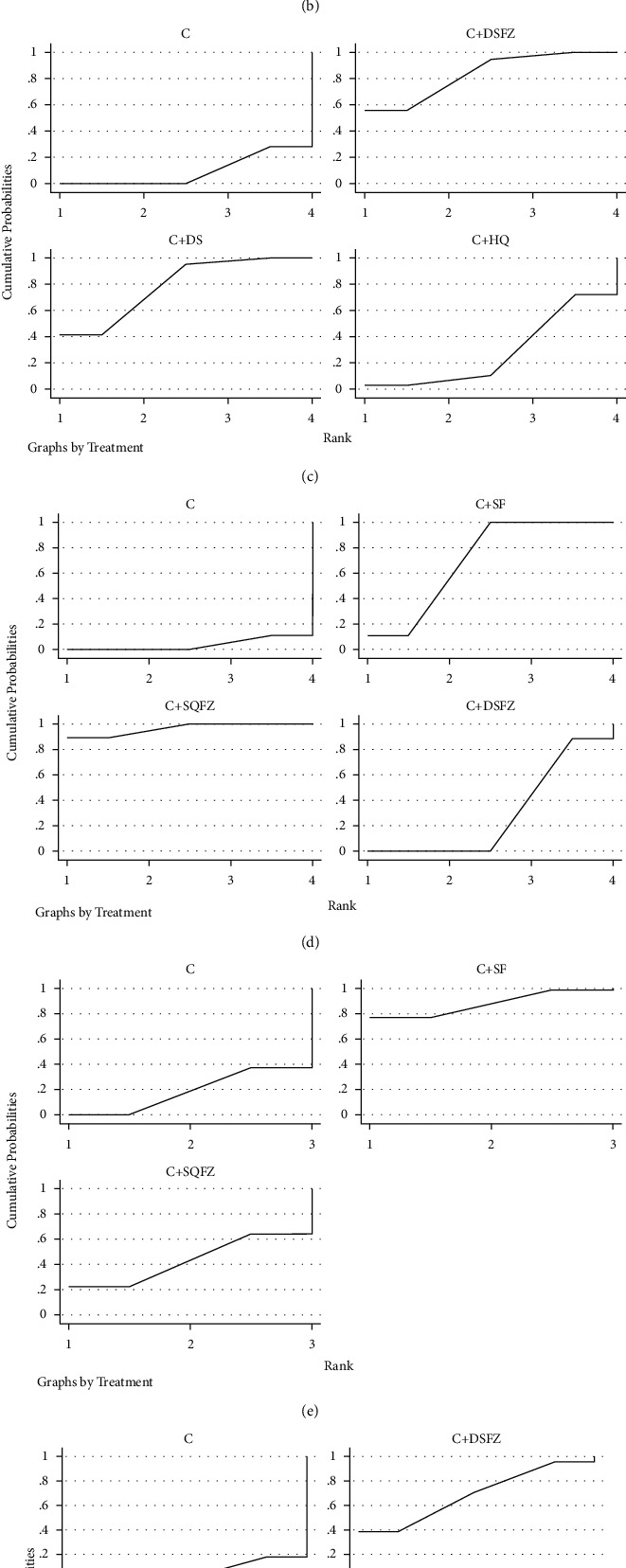
Curve diagram of SUCRA of outcome indicators. (a) Overall response rate; (b) TNF-*α*; (c) IL-6; (d) IL-8; (e) IL-10; (f) recurrence rate; (g) adverse reaction rate. C : Conventional Western medicine, SF:Shenfu injection, SQFZ: Shenqi Fuzheng injection, DSFZ: Danshen powder injection, FFDS: compound Danshen injection, DS: Danshen injection, FFKS: compound Kushen injection, GXN: Guanxinning injection, HH:Safflower injection, HQ: *Astragalus* injection, SM1 : Shenmai injection, CWJ: Acanthopanax injection, SM2 : Shengmai injection, SXN: Shuxuening injection, XD:Xiangdan injection, XSTF: Xuesaitong powder injection, YXC: Houttuynia injection, and DG: Angelica injection.

*(3) Network Meta-analysis*. Thirty-seven pieces of literature reported overall response rates involving 17 CHIs. Network comparison was conducted in 17 CHIs, yielding a total of 136 pairwise comparisons, nine of which were statistically significant. Compared with traditional Western medicine, OR and 95% CI of traditional west medicine integrated with Xiangdan injection, traditional Western medicine integrated with Shengmai injection, traditional Western medicine integrated with Danshen injection, traditional Western medicine integrated with Danshen powder injection, traditional Western medicine integrated with Xuesaitong powder injection, traditional Western medicine integrated with Shuxuening injection, and traditional Western medicine integrated with *Astragalus* injection were 12.25 and [1.50, 99.80], 4.91 and [1.74, 13.85], 4.24 and [2.81, 6.40],3.65 and [2.09, 6.37], 3.47 and [1.13, 10.68], 3.41 and [1.72, 6.76], and 3.16 and [1.65, 6.06], respectively. The specific results are shown in Figure 5.

**Figure 6:**
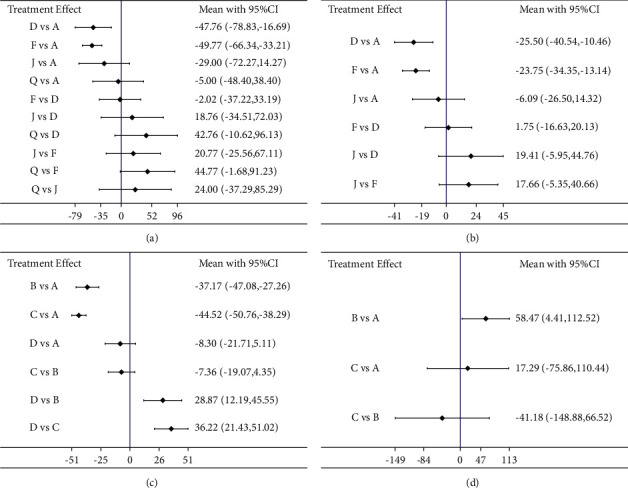
Pairwise comparison forest graph of outcome indicators. (a)TNF-*α*; (b) IL-6; (c) IL-8; (d) IL-10. A : Conventional Western medicine, B : conventional Western medicine + Shenfu injection, C : conventional Western medicine + Shenqi Fuzheng injection, D : conventional Western medicine + Danshen powder injection, E : conventional Western medicine + compound Danshen injection, F : conventional Western medicine + Danshen injection, G : conventional Western medicine + compound Kushen injection, H:conventional Western medicine + Guanxinning injection, I : conventional Western medicine + Safflower injection, J : conventional Western medicine + *Astragalus* injection, K : conventional Western medicine + Shenmai injection, L : conventional Western medicine + Acanthopanax injection, M : conventional Western medicine + Shengmai injection, N : conventional Western medicine + Shuxuening injection, O : conventional Western medicine + Xiangdan injection, P : conventional Western medicine + Xuesaitong powder injection, and Q : conventional Western medicine + Houttuynia injection.

*(4) SUCRA Probability Ranking*. In accordance with the area under the curve diagram of SUCRA (Figure 6), the overall response rates of 17 CHI and traditional west medicine were ranked probabilistically from high to low as follows: C + XD (83.8%) > C + SM1 (78.8%) > C + SM2 (66.1%) > + C + DS (62.9%) > C + YXC (62.1%) > C + FFDS (58.7%) > C + FFKS (57.1%) > C + DSFZ (53.8%) > C + XSTF (50.9%) > C + SXN (50.0%) > C + HQ (46.0%) > C + HH (42.6%) > C + SF (42.6%) > C + GXN (40.0%) > C + SQFZ (34.6%) > C + CWJ (14.8%) > C (5.1%).

#### 3.4.2. Inflammatory Factors

*(1) Tumor necrosis Factor-Alpha (TNF-α) Evidence Network*. 11 pieces of literature reported TNF-*α*, involving 4 CHI treatment regimens. The dot size indicates the sample size using the intervention, the line thickness represents the number of RCTs using the two-point treatment intervention, all 4 CHIs denote direct comparisons, and there is no closed-loop formation. The network evidence of the TNF-*α* is exhibited in Figure 3.

**Figure 7:**
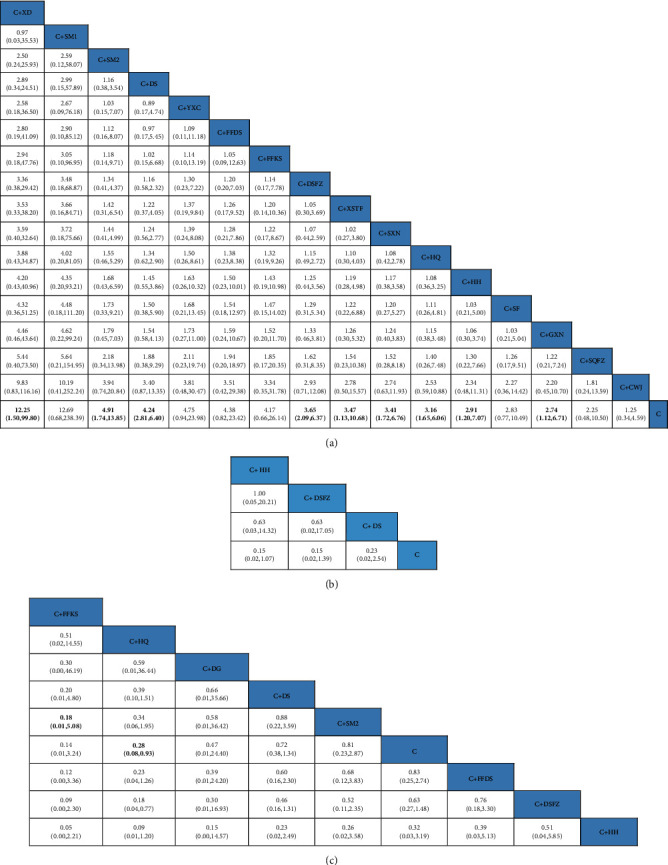
(a–c) Results from the NMA showing the effect of each of the interventions. (a) ORs with 95% CIs of the overall response rates. (b) ORs with 95% CIs of the recurrence rate. (c)ORs with 95% CIs of the adverse reactions. The values in bold font represent statistically significant differences.

 
*Publication Bias*. The funnel plot of this study revealed that most of the scatter points were located on both sides of the vertical line. They were basically symmetrical and may have a certain degree of publication bias. The funnel plot of the TNF-*α* of 4 CHIs integrated with traditional Western medicine for treating UC is presented in Figure 4.
 
*Network Meta-analysis*. 11 pieces of literature reported TNF-*α* involving 4 CHIs. Network comparison was conducted in 4 CHIs, yielding a total of 20 pairwise comparisons, two of which were statistically significant. Compared with traditional Western medicine, MD and 95% CI of traditional Western medicine integrated with Danshen powder injection and traditional Western medicine integrated with Danshen injection, were −47.76 and [−78.83, −16.69] and −49.77 and [−66.34, −33.21], respectively. The specific results are shown in Figure 7(a).
 
*SUCRA Probability Ranking*. In accordance with the area under the curve diagram of SUCRA (Figure 5), the reduced TNF-*α* rate of 4 CHI and traditional west medicine were ranked probabilistically from high to low as follows: C + DS (83.2%) > C + DSFZ(78.8%) > C + HQ (53.1%) > C + YXC (22.4%) > C (12.7%).

#### 3.4.3. Interleukin-6 (IL-6)

*(1) Evidence Network*. Thirty-seven pieces of literature reported IL-6, involving 3 CHI treatment regimens. The dot size indicates the sample size using the intervention, the line thickness represents the number of RCTs using the two-point treatment intervention, all 3 CHIs denote direct comparisons, and there is no closed-loop formation. The network evidence of the IL-6 is exhibited in Figure 3.

*(2) Publication Bias*. The funnel plot of this study revealed that most of the scatter points were located on both sides of the vertical line. They were basically symmetrical and may have a certain degree of publication bias. The funnel plot of the IL-6 of 3 CHIs integrated with traditional Western medicine for treating UC is presented in Figure 4.

*(3) Network Meta-analysis*. 7 pieces of literature reported IL-6, involving 3 CHIs. Network comparison was conducted in 3 CHIs, yielding a total of 12 pairwise comparisons, two of which were statistically significant. Compared with traditional west medicine, MD and 95% CI of traditional Western medicine integrated with Danshen powder injection and traditional west medicine integrated with Danshen injection, were−25.50 and [−40.54, −10.46] and −23.75 and [−34.35, −13.14], respectively. The specific results are shown in Figure 6.

*(4) SUCRA Probability Ranking*. In accordance with the area under the curve diagram of SUCRA (Figure 5), the reduced IL-6pa rate of 3 CHI and traditional west medicine were ranked probabilistically from high to low as follows: C + DSFZ (83.7%) > +C + DS (78.8%) > C + HQ (28.6%) > C (9.3%).

#### 3.4.4. Interleukin-8 (IL-8) Evidence Network

9 pieces of literature reported IL-8, involving 3 CHI treatment regimens. The dot size indicates the sample size using the intervention, the line thickness represents the number of RCTs using the two-point treatment intervention, all 3 CHIs denote direct comparisons, and there is no closed-loop formation. The network evidence of the IL-8 is exhibited in Figure 3.

*(1) Publication Bias*. The funnel plot of this study revealed that most of the scatter points were located on both sides of the vertical line. They were basically symmetrical and may have a certain degree of publication bias. The funnel plot of the IL-8 of 3 CHIs integrated with traditional west medicine for treating UC is presented in Figure 4.

*(2) Network Meta-analysis*. 9 pieces of literature reported IL-8, involving 3 CHIs. Network comparison was conducted in 3 CHIs, yielding a total of 12 pairwise comparisons, two of which were statistically significant. Compared with traditional Western medicine, MD and 95% CI of traditional Western medicine integrated with Shenfu injection and traditional west medicine integrated with Shenqi Fuzheng injection, were −37.17 and [−47.08, −27] and −44.52 and [−50.76, −38.29], respectively. The specific results are shown in Figure 6.

*(3) SUCRA Probability Ranking*. In accordance with the area under the curve diagram of SUCRA (Figure 5), the reduced IL-8 rate of 3 CHI and traditional Western medicine were ranked probabilistically from high to low as follows: C + SQFZ (96.3%) > C + SF (70.2%) > +C + DSFZ (29.5%) > C (3.8%).

#### 3.4.5. Interleukin-10 (IL-10)

*(1) Evidence Network*. 4 pieces of literature reported IL-10, involving 2 CHI treatment regimens. The dot size indicates the sample size using the intervention, the line thickness represents the number of RCTs using the two-point treatment intervention, all 2 CHIs denote direct comparisons, and there is no closed-loop formation. The network evidence of IL-10 is exhibited in Figure 3.

*(2) Publication Bias*. The funnel plot of this study revealed that most of the scatter points were located on both sides of the vertical line. They were basically symmetrical and may have had a certain degree of publication bias. The funnel plot of the IL-10 of 2 CHIs integrated with traditional Western medicine for treating UC is presented in Figure 4.

*(3) Network Meta-analysis*. 4 pieces of literature reported IL-10, involving 2 CHIs. Network comparison was conducted in 2 CHIs, yielding a total of 6 pairwise comparisons, two of which were statistically significant. Compared with traditional Western medicine, the MD and 95% CI of traditional Western medicine integrated with Shenfu injection were 4.41 and [112, 52]. The specific results are shown in Figure 6.

*(4) SUCRA Probability Ranking*. In accordance with the area under the curve diagram of SUCRA (Figure 5), the increased IL-10 rate of 3 CHI and traditional Western medicine were ranked probabilistically from high to low as follows: C + SF (87.8%) > C + SQFZ (43.4%) >  + C (18.8%).(43.4%) >  + C (18.8%).

#### 3.4.6. Recurrence Rate Evidence Network

4 pieces of literature reported a recurrence rate, involving 3 CHI treatment regimens. The dot size indicates the sample size using the intervention, the line thickness represents the number of RCTs using the two-point treatment intervention, all 3 CHIs denote direct comparisons, and there is no closed-loop formation. The network evidence of the recurrence rate is exhibited in Figure 3.

*(1) Publication Bias*. The funnel plot of this study revealed that most of the scatter points were located on both sides of the vertical line. They were basically symmetrical and may have had a certain degree of publication bias. The funnel plot of the recurrence rate of 3 CHIs integrated with traditional Western medicine for treating UC is presented in Figure 4.

*(2) Network Meta-analysis*. 4 pieces of literature reported a recurrence rate, involving 3 CHIs. A network comparison was conducted in 3 CHIs, yielding a total of 6 pairwise comparisons. The results showed that no significant difference was identified in the recurrence rate between the 4 interventions. The specific results are shown in Figure 7(b).

*(3) SUCRA Probability Ranking*. In accordance with the area under the curve diagram of SUCRA (Figure 5), the reduced recurrence rate of 3 CHI and traditional Western medicine were ranked probabilistically from high to low as follows: C + HQ (69.6%) > C + DSFZ (68.6%) > C + DS (55.4%) > C (6.4%).

#### 3.4.7. Incidence of Adverse Reactions

*(1) Evidence Network*. 4 pieces of literature reported an incidence of adverse reactions involving 8 CHI treatment regimens. The dot size indicates the sample size using the intervention, the line thickness represents the number of RCTs using the two-point treatment intervention, all 8 CHIs denote direct comparisons, and there is no closed-loop formation. The network evidence of the incidence of adverse reactions is exhibited in Figure 3.

*(2) Publication Bias*. The funnel plot of this study revealed that most of the scatter points were located on both sides of the vertical line. They were basically symmetrical and may have had a certain degree of publication bias. The funnel plot of the incidence of adverse reactions of 8 CHIs integrated with traditional Western medicine for treating UC is presented in Figure 4.

*(3) Network Meta-analysis*. 4 pieces of literature reported an incidence of adverse reactions involving 8 CHIs. Network comparison was conducted in 8 CHIs, yielding a total of 36 pairwise comparisons, two of which were statistically significant. Compared with traditional Western medicine, the OR and 95% CI of traditional Western medicine integrated with *Astragalus* injection and traditional Western medicine integrated with Danshen powder injection were 0.28 and [0.08, 0.93] and 0.18 and [0.04, 0.77], respectively. The specific results are shown in Figure 7(c).

*(4) SUCRA Probability Ranking*. In accordance with the area under the curve diagram of SUCRA (Figure 5), the reduced Incidence of adverse reactions of 8 CHI and traditional Western medicine were ranked probabilistically from high to low as follows: C + FFKS (83.4%) > C + HQ (83%) > C + DG (59.1%) > C + DS (58.4%) > C + SM2 (50.4%) > C (41.5%) > C + FFDS (35.3%) > C + DSFZ (23%)>C + HH (15.8%).

## 4. Discussion

With the increasing number of published clinical studies of CHI for UC, clinical reports of different CHIs combined with conventional therapies for the adjuvant treatment of UC are also on the rise. However, no definitive conclusion has been reached as to which of the different CHI adjuvant therapies has the best adjuvant effect on UC. We believe that traditional meta-analyses limited only by a two-by-two comparison no longer provide valid methodological support for the selection of the optimal intervention for the treatment of CSR. In contrast, network meta-analysis allows for comparisons between multiple interventions. Therefore, this study is the first to compare the efficacy and safety of different CHI adjuvant treatments for UC using a network meta-analysis based on a frequency-based framework, with the aim of synthesizing direct versus indirect comparisons and providing a more credible evidence-based medical basis for the clinical treatment of UC.

The ranking results demonstrated that regarding the probability of overall clinical response rate, the efficacy ranking was Xiangdan injection > Shenmai injection > Shengmai injection > Danshen injection > Houttuynia injection > Compound Danshen injection > Compound Kushen injection > Danshen powder injection > Xuesaitong powder injection > Shuxuening injection > *Astragalus* injection > Safflower injection > Shenfu injection > Guanxinning injection > Shenqi Fuzheng injection > Acanthopanax senticosus injection > traditional Western medicine; in terms of the reduced TNF-*α*levels, the probability ranking was Danshen injection > Danshen powder injection > *Astragalus* injection > Houttuynia injection > traditional Western medicine; concerning the reduced IL-6 levels, the probability ranking was Danshen powder injection > Danshen injection > *Astragalus* injection > traditional Western medicine; for the reduced IL-8 levels, the probability ranking was Shenqi Fuzheng Injection > Shenfu Injection > Danshen Powder Injection > traditional Western medicine; with respect to the increased IL-10 levels, the probability ranking was Shenfu injection > Shenqi Fuzheng injection > traditional Western medicine; as for the reduced recurrence rate, the probability ranking was *Astragalus* injection > Danshen powder injection > Danshen injection > traditional west medicine; respecting the reduced adverse reactions, the probability ranking was compound Kushen injection > *Astragalus* injection > Angelica sinensis injection > Danshen injection > Shengmai injection > traditional west medicine > compound Danshen injection > Danshen powder injection > Safflower injection.

The results suggested that Xiangdan injection, Shenmai injection, Shengmai injection, and Danshen injection integrated with traditional Western medicine treatment possessed the greatest possibility to be the optimal regimen. In the multiple-group comparison of the above overall response rate, inflammatory factors, recurrence rate, and outcome measures of the incidence of adverse reactions, the performance of traditional Western medicine alone ranked low. Thus, a combination of traditional Chinese and Western medicine was superior to traditional Western medicine alone.

In summary, Xiangdan injection, Shenmai injection, Shengmai injection, and Danshen injection integrated with traditional Western medicine treatment ranked first regarding effective rate. Besides, it was most likely to be the optimal regimen among the above interventions included. The top interventions could be selected for patients with different goals.

Through extensive comparisons with previous studies, we found that the top four herbal injections in terms of efficacy have had a certain number of relevant experimental animal studies published. These studies are expected to add credibility to the findings of the current study from the perspective of basic research and shed more light on the mechanism of action of CHI for UC.

The main ingredient of Xiangdan injection is the extract of the Chinese herbal medicine Dan, which is involved in descending incense. Salvia miltiorrhiza has the function of activating blood circulation, relieving pain, clearing the heart, relieving irritation, cooling the blood, and eliminating carbuncles. Radix *Rehmanniae* has the ability to resolve blood stasis and stop bleeding, regulate Qi, and relieve pain. A large number of animal experimental studies have shown [62–65] that cryptotanshinone, tanshinone IIA, and dihydrotanshinone I, the main natural compound components in Xiangdan injection, intervene in mice with ulcerative colitis to accelerate the speed of microcirculatory blood flow and improve mesenteric microcirculation, which is conducive to accelerating the repair of injured intestinal mucosa and promoting the healing of ulcerated surfaces.

Ginseng and maitake injection is mainly composed of red ginseng and maitake, which have the function of tonifying qi and consolidating essence, nourishing yin and generating fluid. A considerable number of published animal experiments have shown [66–68] that red ginseng extract, the main ingredient in ginseng and wheat injection, can significantly improve the structure of the intestinal microbiota of rats with ulcerative colitis and alleviate the symptoms of ulcerative colitis in vivo. And it can alleviate macroscopic lesions such as shortened colons, blood in stool and weight loss in mice with dextran sodium sulfate (DSS)-induced ulcerative colitis.

The main ingredient of Shengvei injection is the extract of Chinese herbal medicine, Red Ginseng, Mai Dong, and Wu Wei Zi, which has the effect of benefiting Qi and nourishing Yin, restoring the pulse and fixing detachment. An animal experimental study by Juan Lu, Yue Yu et al. [69] showed that raw vein injection had a protective effect on the intestinal mucosa of mice and was able to significantly improve the survival rate in a mouse model of inflammation.

The main ingredient of Danshen injection is the extract of the Chinese medicine Danshen. It has the efficacy of activating blood circulation, removing blood stasis, opening the veins, and nourishing the heart.

A related animal experimental study [70] showed that the combination of total phenolic acid of Danshen stem leaves, the main component of Danshen injection, and tanshinone significantly improved the symptoms of ulcerative colitis in mice by inhibiting the TLR4/PI3K/AKT/mTOR signaling pathway and exerting a protective effect on the intestinal mucosa of mice with dextran sulfate sodium (DSS)-induced ulcerative colitis.

## 5. Limitation

In this study, the safety and efficacy of various CHIs for treating UC were compared by network meta-analysis, providing a certain reference for clinical practice. However, there are still some limits here. First, the methodological quality of the involved literature was small on the whole. Among the 42 pieces of involved literature, 31 did not specify the random sequence generation method. None of the literature employed a random allocation concealment method. Besides, selectivity bias may exist during subject selection. None of the literature involved the blind method between subjects and interventionalists, and only one study used pathological result double-blind for the assessment of the results, making it a potential source of bias in the assessment of results. Four pieces of literature had dropouts/withdrawals. Moreover, patient age, drug dosage, and treatment process differed between the literature with some clinical heterogeneity. Additionally, there may be some small sample effects, leading to publication bias in the study. Combined with related basic research, we found that there are still some gaps in the research of direct intervention of CHI in UC animal experiments. Further relevant in vivo or in vitro model validation is pending. At the same time, it is recommended that more standardized randomized controlled double-blind trials with good quality, large samples, and multicenter participation are needed in the future to provide a stronger basis for the safety and efficacy of integrating CHI with conventional drugs for UC.

## 6. Conclusion

The result of the network meta-analysis indicated that CHI integrated with traditional drugs is likely to be effective for treating UC. For treating UC, the overall response rate of CHI integrated with traditional drugs, which could better reduce TNF-*α*, IL-6, and IL-8 levels and better increase IL-10 levels, was higher than that of traditional treatment. Besides, CHI integrated with traditional Western medicine can better reduce the incidence of adverse reactions, and there is no significant difference in the recurrence rate. In our involved literature, Xiangdan injection, Shenmai injection, Shengmai injection, and Danshen injection may be the most effective CHI. Nevertheless, more high-quality, large sample, and multi-center randomized controlled double-blind trials are still required for research confirmation.

## Data Availability

All the data in this study are available within the manuscript.

## Additional Points

The authors have completed the PRISMA reporting checklist.

## Supplementary Materials

Supplementary Materials Literature search strategy. Supplementary Table 2: basic situation of the included studies. Supplementary Table 3: checklist of the PRISMA extension for network meta-analysis.
