# Supplementary material for: Chinese Herb Injections in the Adjuvant Treatment for Ulcerative Colitis: A Network Meta-Analysis of Randomized Controlled Trials
Source: Evid Based Complement Alternat Med. 2022 Apr 22;2022:3166416. doi: 10.1155/2022/3166416 (PMC9054452; doi:10.1155/2022/3166416)
Supplement: Supplementary Materials — Literature search strategy. Supplementary Table 2: basic situation of the included studies. Supplementary Table 3: checklist of the PRISMA extension for network meta-analysis. [file 3166416.f1.docx]

**Supplementary Table 1.Literature search strategy**

| **Database** | **Search strategy** |
| --- | --- |
| **Pubmed** | ((Ulcerative colitis[Title/Abstract]) OR (Nonspecific ulcerative colitis[Title/Abstract])) AND (([Shenfu Injection[Title/Abstract]) OR (Shenqi Fuzheng Injection[Title/Abstract]) OR (Danshen powder injection[Title/Abstract]) OR (Compound Danshen Injection[Title/Abstract]) OR (Danshen Injection[Title/Abstract])) OR (Compound Kushen Injection[Title/Abstract]) OR (Guanxinning Injection[Title/Abstract]) OR (Safflower injection[Title/Abstract]) OR (Astragalus Injection[Title/Abstract] )OR (Shenmai Injection[Title/Abstract] )OR (Acanthopanax Injection[Title/Abstract]) OR (Shengmai injection[Title/Abstract]) OR (Shuxuening Injection[Title/Abstract]) OR (Xiangdan injection[Title/Abstract]) OR (Xuesaitong powder injection[Title/Abstract]) OR (Houttuynia injection[Title/Abstract]) OR (Angelica injection[Title/Abstract])) |
| **Embase** | 1# Ulcerative colitis:ab,ti OR Nonspecific ulcerative colitis :ab,ti  2# Shenfu Injection:ab,ti OR Shenqi Fuzheng Injection:ab,ti OR Danshen powder injection:ab,ti OR Compound Danshen Injection:ab,ti OR Danshen Injection:ab,ti OR Compound Kushen Injection:ab,ti OR Guanxinning Injection:ab,ti OR Safflower injection:ab,ti OR Astragalus Injection:ab,ti OR Shenmai Injection:ab,ti OR Acanthopanax Injection:ab,ti OR Shengmai injection:ab,ti OR Shuxuening Injection:ab,ti OR Xiangdan injection:ab,ti OR Xuesaitong powder injection :ab,ti OR Houttuynia injection:ab,ti  OR Angelica injection :ab  3# 1#AND 2# |
| **The Cochrane Library** | Ulcerative colitis or Nonspecific ulcerative colitis in Title Abstract Keyword AND Shenfu Injection or Shenqi Fuzheng Injection or Danshen powder injection or Compound Danshen Injection or Danshen Injection or Compound Kushen Injection or Guanxinning Injection or Safflower injection or Astragalus Injection or Shenmai Injection or Acanthopanax Injection or Shengmai injection or Shuxuening Injection or Xiangdan injection or Xuesaitong powder injection or Houttuynia injection or Angelica injection in Title Abstract Keyword - (Word variations have been searched) |
| **CNKI** | (SU=kui yang xing jie chang yan OR SU=fei te yi xing kui yang xing jie chang yan) AND (SU=shen fu OR SU=shen qi fu zheng OR SU=dan shen fen OR SU=fu fang dan shen OR SU=dan shen OR SU=fu fang ku shen OR SU=guan xin ning OR SU=hong hua OR SU=huang qi OR SU=shen mai OR SU=ci wu jia OR SU=sheng mai OR SU=shu xue ning OR SU=xiang dan OR SU=xue se tong OR SU=yu xing cao OR SU=dang gui OR SU=zhu she ji OR SU=zhu she yao OR SU=zhu she ye OR SU=zhen ji)  (SU refers to theme） |
| **Wanfang database** | Theme:("kui yang xing jie chang yan"+"fei te yi xing kui yang xing jie chang yan")*Theme:("shen fu"+"shen qi fu zheng"+"dan shen fen"+"fu fang dan shen"+"dan shen"+"fu fang ku shen"+"guan xin ning"+"hong hua"+"huang qi"+"shen mai"+"ci wu jia"+"sheng mai"+"shu xue ning"+"xiang dan"+"xue se tong"+"yu xing cao"+"dang gui"+"zhu she ji"+"zhu she yao"+"zhu she ye"+"zhen ji") |
| **VIP** | (M=kui yang xing jie chang yan OR M=fei te yi xing kui yang xing jie chang yan) AND (M=shen fu OR M=shen qi fu zheng OR M=dan shen fen OR M=fu fang dan shen OR M=dan shen OR M=fu fang ku shen OR M=guan xin ning OR M=hong hua OR M=huang qi OR M= shen mai OR M=ci wu jia OR M=sheng mai OR M=shu xue ning OR M=xiang dan OR M=xue se tong OR M=yu xing cao OR M=dang gui OR M=zhu she ji OR M=zhu she yao OR M=zhu she ye OR M=zhen ji）  (M refers to title and keyword.) |
| **CBM** | [("kui yang xing jie chang yan"[All fields: smart] OR "fei te yi xing kui yang xing jie chang yan"[All fields: smart]) AND ( "shen fu"[All fields: smart] OR "shen qi fu zheng"[All fields: smart] OR "dan shen fen"[All fields: smart] OR "fu fang ku shen"[All fields: smart] OR "guan xin ning"[All fields: smart] OR "hong hua"[All fields: smart] OR "huang qi"[All fields: smart]](http://www--sinomed--ac--cn--http.sinomed.gzzyy.qfclo.com:2222/javascript:toDoRelimitSearch();) OR "shen mai"[All fields: smart] OR "ci wu jia"[All fields: smart] OR "sheng mai"[All fields: smart] OR "shu xue ning"[All fields: smart] OR "xiang dan"[All fields: smart] OR "xue se tong"[All fields: smart] OR "yu xing cao"[All fields: smart] OR "dang gui"[All fields: smart] OR "zhu she ji"[All fields: smart] OR "zhu she yao"[All fields: smart] OR "zhu she ye"[All fields: smart] OR "zhen ji"[All fields: smart]） |

**Supplementary Table 2.Basic situation of the included studies**

| Study | Sample size | | Male /Female | Age (mean or range) | | Course of disease（mean or range） | | Intervention of  Experimental group | Intervention of  Control group | Course of treatment/d | Outcome indicators |
| --- | --- | --- | --- | --- | --- | --- | --- | --- | --- | --- | --- |
|  | E | C |  | E | C | E | C |  |  |  |  |
| Wang（2002）^[21]^ | 38 | 32 | 44/26 | - | - |  |  | Shenfu Injection+Sulfasalazine | Sulfasalazine | 15 | （1） |
| Zhao(2015)^[22]^ | 25 | 25 | 23/28 | - | - | - | - | Shenqi Fuzheng Injection＋Sulfasalazine | Sulfasalazine | 14 | （1） |
| Li（2019)^[23]^ | 51 | 51 | 60/42 | - | - | - | - | Danshen powder injection＋Mesalamine | Mesalamine | 56 | （1)（2）（4） |
| Wang2018 ^[24]^ | 60 | 60 | 57/63 | 50.2 ± 9.8 | 51.0 ± 10. 7 | 5.8 ± 1.2 | 5.6 ± 1.1 | Danshen powder injection＋Mesalamine | Mesalamine | 18 | （1）（2）（3）（4） |
| Zhang2008^[25]^ | 27 | 27 | 29/25 | 33 .2 | 32 .8 | - | - | Compound Danshen Injection+Sulfasalazine | Sulfasalazine | 42 | （1）（4） |
| Huang2005^[26]^ | 50 | 50 | 53/47 | 36.8±11.3 | 36.6±12.7 | - | - | Danshen Injection+Sulfasalazine | Sulfasalazine | 30 | （1）（4） |
| Yao2016^[27]^ | 30 | 30 | 42/18 | 48.1±7.9 | 47.9±8.1 | 3.1±0.6 | 3.2±0.7 | Danshen Injection+Ornidazole Sodium Chloride Injection | Ornidazole Sodium Chloride Injection | 28 | （1）（4） |
| Liu2019^[28]^ | 32 | 32 | 35/29 | 40.4±8.5 | 39.9±6.5 | - | - | Danshen Injection+Mesalamine+Compound lactic acid bacteria capsules | Mesalamine+Compound lactic acid bacteria capsules | 28 | （4） |
| Yang2017^[29]^ | 47 | 46 | 49/44 | 34.20±6.46 | 33.17±6.50 | 4.90±3.09 | 4.88±3.12 | Danshen Injection+Mesalamine+Sulfasalazine | Mesalamine+Sulfasalazine | 180 | （4） |
| Zhu2019^[30]^ | 49 | 49 | 57/41 | 37.4±5.7 | 37.6±5.8 | - | - | Danshen Injection+Mesalamine | Mesalamine | 28 | （2）（4） |
| Chen2018^[31]^ | 39 | 39 | 42/36 | 47.13±6.61 | 46.48±6.34 | - | - | Danshen Injection+Mesalamine | Mesalamine | 14 | （1）（2） |
| Wang2017^[32]^ | 45 | 45 | 51/39 | 43.5±2.9 | 43.4±2.8 | - | - | Danshen Injection+Mesalamine | Mesalamine | 28 | （1）（2）（4） |
| Yang2017^[33]^ | 30 | 30 | 33/27 | 40.2±8.8 | 39.3±6.7 | - | - | Danshen Injection+Mesalamine | Mesalamine | 28 | （1） |
| Ma2013^[34]^ | 74 | 73 | 82/65 | 40.2±13.2 | 39.9±14.4 | - | - | Danshen Injection+Sulfasalazine | Sulfasalazine | 30 | （1） |
| Liu2003^[35]^ | 31 | 30 |  | - | - | - | - | Angelica injection+Mesalazine | Mesalazine | 21 | （4） |
| Tian2011^[36]^ | 17 | 15 | 17/15 | 32±10.6 | 42±13 | - | - | Compound Kushen Injection+Metronidazole+  Dexamethasone | Metronidazole+  Dexamethasone | 10 | （1） |
| Zhang2010^[37]^ | 61 | 59 | 68/52 | 40.89±8.90 | 39.76±8.52 | - | - | Guanxinning Injection+Mesalamine | Mesalamine | 14 | （1） |
| Wu2015^[38]^ | 60 | 60 | 74/46 | 40.2±5.1 | 39.5±4.7 | - | - | Safflower injection+Sulfasalazine | Sulfasalazine | 15 | （1） |
| Yan2011^[39]^ | 32 | 32 | 38/26 | 40.67±9.12 | 39.84±9.53 | - | - | Safflower injection+Sulfasalazine | Sulfasalazine | 15 | （1） |
| Jia2014^[40]^ | 14 | 14 | 18/10 | - | - | - | - | Astragalus Injection+Dexamethasone+Metronidazole+Montmorillonite powder | Dexamethasone+Metronidazole+Montmorillonite powder | 28 | （1)（3） |
| Li(2005)^[41]^ | 76 | 75 | - | - | - | - | - | Astragalus Injection+Sulfasalazineor Mesalazine | Sulfasalazine + Mesalazine | 28 | （1） |
| Liu(2011)^[42]^ | 42 | 39 | 45/36 | - | - | - | - | Astragalus Injection+Mesalamine | Mesalamine | 30 | （1）（3） |
| Li(2013)^[43]^ | 48 | 48 | 60/36 | 43.6±8.9 | 42 5±9.4 | 64.7±12.6 | 65.4±13.9 | Astragalus Injection+Mesalamine | Mesalamine | 30 | （1） |
| Hu(2020)^[44]^ | 53 | 53 | 57/49 | 42.59±5.19 | 43.71±5.27 | 4.19±3.29 | 4.79±3.41 | Astragalus Injection+Mesalamine | Mesalamine | 15 | （1）（2） |
| Li(2019)^[45]^ | 37 | 37 | 52/22 | 43.2±9.3 | 45.2±9.4 | 3.17±1.23 | 3.15±1.33 | Shenmai Injection+Sulfasalazine | Sulfasalazine | E：20~28；C：60 | （1） |
| Xu(2020)^[46]^ | 44 | 44 | 49/39 | 39.50 ± 3.50 | 39.00±3.50 | 5.50±1.50 | 5.00±1.50 | Danshen Injection+Mesalamine | Mesalamine | 30 | （1）（2） |
| Chen(2012)^[47]^ | 32 | 32 | 37/27 | 35.6±11.3 | 36.8±10.4 | 2.9±1.4 | 3.2±1.7 | Acanthopanax Injection+Sulfasalazine+Hydrocortisone | Sulfasalazine+Hydrocortisone | 40 | （1） |
| Deng(2016)^[48]^ | 55 | 55 | 48/62 | 58.3±8.2 | 57.6±7.5 | 4.8±1.4 | 4.5 ±1.3 | Danshen Injection+Mesalamine | Mesalamine | 28 | （1）（2）（3） |
| Li(2016)^[49]^ | 60 | 60 | 68/52 | - | - | - | - | Danshen Injection+Mesalamine | Mesalamine | 28 | （1）（2） |
| Sun(2019)^[50]^ | 43 | 43 | 48/38 | 45.6±1.2 | 45.4±1.3 | 2.3±0.4 | 2.4±0.3 | Danshen Injection+Mesalamine | Mesalamine | 30 | （1） |
| Zhu(2018)^[51]^ | 27 | 27 | 31/23 | 38.53±10.37 | 39.84±9.68 | 2.51±1.74 | 2.75±1.28 | Danshen Injection+Mesalamine | Mesalamine | 28 | （1）（2） |
| Xu(2008)^[52]^ | 50 | 50 | 52/48 | 38.4±6.15 | 38.4±6.15 | 2.76±1.36 | 2.95±1.42 | Shengmai injection+Mesalamine | Mesalamine | 14 | （1） |
| Liang(2019)^[19]^ | 33 | 33 | 37/29 | 37.8±6.4 | 38.2±6.6 | 2.8± 0.5 | 2.9±0.6 | Shengmai injection+Mesalamine | Mesalamine | 14 | （1） |
| Re(2017)^[53]^ | 40 | 40 | 49/31 | 43. 5±9. 12 | 44. 5±9. 03 | - | - | Shuxuening Injection+Mesalamine | Mesalamine | 15 | （1） |
| Xie(2014)^[54]^ | 47 | 44 | 46/45 | 47 ± 10 | 50 ± 7 | 3. 00 ± 0. 23 | 4. 78 ± 0. 25 | Shuxuening Injection+ Mesalamine | Mesalamine | 14 | （2） |
| Liu(2015)^[55]^ | 55 | 45 | 69/31 | 41 | 34 | - | - | Shuxuening Injection+Mesalamine | Mesalamine | 84 | （1） |
| Wan(2012)^[56]^ | 27 | 27 | 26/28 | - | - | - | - | Shuxuening Injection+Mesalamine+Glutamine+Montmorillonite powder | Mesalamine+Glutamine+Montmorillonite powder | 14 | （1） |
| Zhang(2006)^[57]^ | 50 | 50 | 66/34 | - | - | - | - | Xiangdan injection+Cameazole+ Dexamethasone | Cameazole+ Dexamethasone | 28 | （1） |
| Tang(2019)^[58]^ | 34 | 22 | 40/16 | 45.0 | 47.5 | 7.6 | 6.0 | Xuesaitong powder injection+Osalazine+Hydrocortisone Sodium Succinate+Berberine | Osalazine+Hydrocortisone Sodium Succinate+Berberine | 15 | （1） |
| Zhao(2018)^[59]^ | 40 | 40 | 47/33 | 34.6±9.3 | 34.5±9.7 | 1.6±0.6 | 1.6 ±0.7 | Houttuynia injection+Sulfasalazine | Sulfasalazine | 14 | （1）（2） |
| Liu(2011)^[60]^ | 120 | 120 | 111/89 | 49.53 ± 8.16 | 47.95 ± 7.64 | - | - | Danshen powder injection+Mesalamine | Mesalamine | - | （1） |
| Zhang(2012)^[61]^ | 120 | 120 | 131/109 | 48.52±8.08 | 47.16±7.42 | - | - | Danshen powder injection+Mesalamine | Mesalamine | 56 | （1） |

C: control group; E: experimental group; d: day; (1): the overall response rate; (2): inflammatory factors; (3): recurrence rate; (4):incidence of adverse reactions.

Table S3. Checklist of the PRISMA extension for network meta-analysis.

PRISMA = Preferred Reporting Items for Systematic Reviews and Meta-Analysis; PICOS = population, intervention, comparators, outcomes, study design.

*Text in italics indicates wording specific to reporting of network meta-analyses that has been added to guidance from the PRISMA statement.

**PRISMA NMA Checklist of Items to Include When Reporting A Systematic Review Involving a Network Meta-analysis**

| **Section/Topic** | **Item #** | **Checklist Item** | **Reported on Page #** |
| --- | --- | --- | --- |
| **TITLE** |  |  |  |
| Title | 1 | Identify the report as a systematic review *incorporating a network meta-analysis (or related form of meta-analysis).* | 1 |
|  |  |  |  |
| **ABSTRACT** |  |  | 1 |
| Structured summary | 2 | Provide a structured summary including, as applicable:  **Background:** main objectives  **Methods:** data sources; study eligibility criteria, participants, and interventions; study appraisal; and *synthesis methods, such as network meta-analysis.*  **Results:** number of studies and participants identified; summary estimates with corresponding confidence/credible intervals; *treatment rankings may also be discussed. Authors may choose to summarize pairwise comparisons against a chosen treatment included in their analyses for brevity.*  **Discussion/Conclusions:** limitations; conclusions and implications of findings.  **Other:** primary source of funding; systematic review registration number with registry name. |  |
|  |  |  |  |
| **INTRODUCTION** |  |  |  |
| Rationale | 3 | Describe the rationale for the review in the context of what is already known*, including mention of why a network meta-analysis has been conducted.* | 1-3 |
| Objectives | 4 | Provide an explicit statement of questions being addressed, with reference to participants, interventions, comparisons, outcomes, and study design (PICOS). | 1 |
|  |  |  |  |
| **METHODS** |  |  |  |
| Protocol and registration | 5 | Indicate whether a review protocol exists and if and where it can be accessed (e.g., Web address); and, if available, provide registration information, including registration number. | 4 |
| Eligibility criteria | 6 | Specify study characteristics (e.g., PICOS, length of follow-up) and report characteristics (e.g., years considered, language, publication status) used as criteria for eligibility, giving rationale. *Clearly describe eligible treatments included in the treatment network, and note whether any have been clustered or merged into the same node (with justification).* | 4-5 |
| Information sources | 7 | Describe all information sources (e.g., databases with dates of coverage, contact with study authors to identify additional studies) in the search and date last searched. | 5 |
| Search | 8 | Present full electronic search strategy for at least one database, including any limits used, such that it could be repeated. | Supplementary  Table 1 |
| Study selection | 9 | State the process for selecting studies (i.e., screening, eligibility, included in systematic review, and, if applicable, included in the meta-analysis). | 5-6 |
| Data collection process | 10 | Describe method of data extraction from reports (e.g., piloted forms, independently, in duplicate) and any processes for obtaining and confirming data from investigators. | 5-6 |
| Data items | 11 | List and define all variables for which data were sought (e.g., PICOS, funding sources) and any assumptions and simplifications made. | 5 |
| **Geometry of the network** | **S1** | Describe methods used to explore the geometry of the treatment network under study and potential biases related to it. This should include how the evidence base has been graphically summarized for presentation, and what characteristics were compiled and used to describe the evidence base to readers. | 6-7 |
| Risk of bias within individual studies | 12 | Describe methods used for assessing risk of bias of individual studies (including specification of whether this was done at the study or outcome level), and how this information is to be used in any data synthesis. | 6 |
| Summary measures | 13 | State the principal summary measures (e.g., risk ratio, difference in means). *Also describe the use of additional summary measures assessed, such as treatment rankings and surface under the cumulative ranking curve (SUCRA) values, as well as modified approaches used to present summary findings from meta-analyses.* | 6-7 |
| Planned methods of analysis | 14 | Describe the methods of handling data and combining results of studies for each network meta-analysis. This should include, but not be limited to:   - *Handling of multi-arm trials;* - *Selection of variance structure;* - *Selection of prior distributions in Bayesian analyses; and* - *Assessment of model fit.* | 6-7 |
| **Assessment of Inconsistency** | **S2** | Describe the statistical methods used to evaluate the agreement of direct and indirect evidence in the treatment network(s) studied. Describe efforts taken to address its presence when found. | 6-7 |
| Risk of bias across studies | 15 | Specify any assessment of risk of bias that may affect the cumulative evidence (e.g., publication bias, selective reporting within studies). | 6 |
| Additional analyses | 16 | Describe methods of additional analyses if done, indicating which were pre-specified. This may include, but not be limited to, the following:   - Sensitivity or subgroup analyses; - Meta-regression analyses; - *Alternative formulations of the treatment network; and* - *Use of alternative prior distributions for Bayesian analyses (if applicable).* | 6-7 |
|  |  |  |  |
| **RESULTS†** |  |  |  |
| Study selection | 17 | Give numbers of studies screened, assessed for eligibility, and included in the review, with reasons for exclusions at each stage, ideally with a flow diagram. | 7-8 |
| **Presentation of network structure** | **S3** | Provide a network graph of the included studies to enable visualization of the geometry of the treatment network. | 10 |
| **Summary of network geometry** | **S4** | Provide a brief overview of characteristics of the treatment network. This may include commentary on the abundance of trials and randomized patients for the different interventions and pairwise comparisons in the network, gaps of evidence in the treatment network, and potential biases reflected by the network structure. | 9、13-17 |
| Study characteristics | 18 | For each study, present characteristics for which data were extracted (e.g., study size, PICOS, follow-up period) and provide the citations. | Supplementary  Table 2 |
| Risk of bias within studies | 19 | Present data on risk of bias of each study and, if available, any outcome level assessment. | 10、13、15-18 |
| Results of individual studies | 20 | For all outcomes considered (benefits or harms), present, for each study: 1) simple summary data for each intervention group, and 2) effect estimates and confidence intervals. *Modified approaches may be needed to deal with information from larger networks.* | 10、13、15-18 |
| Synthesis of results | 21 | Present results of each meta-analysis done, including confidence/credible intervals. *In larger networks, authors may focus on comparisons versus a particular comparator (e.g. placebo or standard care), with full findings presented in an appendix. League tables and forest plots may be considered to summarize pairwise comparisons.* If additional summary measures were explored (such as treatment rankings), these should also be presented. | 11-18 |
| **Exploration for inconsistency** | **S5** | Describe results from investigations of inconsistency. This may include such information as measures of model fit to compare consistency and inconsistency models, *P* values from statistical tests, or summary of inconsistency estimates from different parts of the treatment network. | 9、13-18 |
| Risk of bias across studies | 22 | Present results of any assessment of risk of bias across studies for the evidence base being studied. | 10、13、15-18 |
| Results of additional analyses | 23 | Give results of additional analyses, if done (e.g., sensitivity or subgroup analyses, meta-regression analyses*, alternative network geometries studied, alternative choice of prior distributions for Bayesian analyses,* and so forth). | 10、13、15-18 |
|  |  |  |  |
| **DISCUSSION** |  |  |  |
| Summary of evidence | 24 | Summarize the main findings, including the strength of evidence for each main outcome; consider their relevance to key groups (e.g., healthcare providers, users, and policy-makers). | 19-21 |
| Limitations | 25 | Discuss limitations at study and outcome level (e.g., risk of bias), and at review level (e.g., incomplete retrieval of identified research, reporting bias). *Comment on the validity of the assumptions, such as transitivity and consistency. Comment on any concerns regarding network geometry (e.g., avoidance of certain comparisons).* | 21 |
| Conclusions | 26 | Provide a general interpretation of the results in the context of other evidence, and implications for future research. | 21 |
|  |  |  |  |
| **FUNDING** |  |  |  |
| Funding | 27 | Describe sources of funding for the systematic review and other support (e.g., supply of data); role of funders for the systematic review. This should also include information regarding whether funding has been received from manufacturers of treatments in the network and/or whether some of the authors are content experts with professional conflicts of interest that could affect use of treatments in the network. | 22 |
